# Use of herbal medicine by caregivers in the management of children with sickle cell disease in Mulago National Referral Hospital - Uganda

**DOI:** 10.11604/pamj.2021.39.163.20740

**Published:** 2021-07-01

**Authors:** Martin Lubega, Charles Peter Osingada, Phillip Kasirye

**Affiliations:** 1 Department of Nursing, Makerere University, Kampala, Uganda,; 2 Department of Pediatrics, Mulago National Referral Hospital, Kampala, Uganda

**Keywords:** Herbal medicine, use, caregivers

## Abstract

**Introduction:**

Sickle Cell Disease (SCD) is the leading genetic disease in sub-Saharan Africa and therefore remains a global public health threat. Use of complementary and alternative medicines (CAM) most especially herbal medicine (HM) in chronic diseases such as sickle cell disease has widely been reported in Africa where advanced technologies are greatly lacking. Despite a large presence of the sickle cell disease in Uganda, the extent to which herbal medicines are used in management of children with sickle cell disease has not been documented. This study purposed to determine the prevalence of herbal medicine (HM) use and associated factors among caregivers of children with SCD at Mulago National Referral Hospital.

**Methods:**

a total of 384 child caretakers were interviewed in a descriptive cross-sectional quantitative study conducted at the Mulago Sickle cell clinic in March 2019. Enrolment was done consecutively and a structured interviewer administered questionnaire administered to collect data from the caretakers which was managed using SPSS version 23. Multivariate logistic regression was used to identify the factors associated with herbal medicine (HM) use. Factors with p-value <0.05 were regarded significant.

**Results:**

the rate of herbal use was 77.6% (298 of 384 caregivers). At multivariate analysis, the odds of a caregiver who agreed that; HM cures symptoms faster than conventional medicine (CM) were 3 times those who disagreed with this statement (AOR =3.439, 95% CI: 1.447 - 8.176). The odds that a caregiver who agreed that HM has fewer side effects than CM were almost 4 times those that disagreed with this statement (AOR = 3.528, 95% CI: 1.917 -6.494). The odds that a caregiver who agreed that marketing HM through televisions adverts encourages HM use were 4 times those who disagreed with this statement (AOR = 4.185, 95% CI: 2.036 -8.603).

**Conclusion:**

this study reports a high prevalence of HM use among caregivers of children with SCD at Mulago Hospital, in Uganda. The practice is significantly influenced by caretakers´ perception that HM cures symptoms faster than CM, has fewer side effects and that telemarketing has greatly facilitated its use over CM. More effort is therefore needed to encourage clinic attendances and CM use and limit the unfounded TV adverts on HM. There is also need for studies to identify the common HM used so that their efficacy and safety are well studied.

## Introduction

Sickle Cell Disease (SCD) remains a global public health threat with more than 300,000 infants born annually [1,2]. However, more than 75% of the global burden of sickle cell anemia (SCA) occurs in sub-Saharan Africa [3], where 50% to 90% of infants born with SCD die before their fifth birthday [4]. Globally, various researchers have reported that use of Complementary and Alternative medicines (CAM) is exceptionally high among children with chronic diseases like asthma, epilepsy and SCD [5,6]. According to WHO, 80% of the world´s population relies on CAM for therapy either as the mainstay of health care delivery or serving as a complement to it [7]. Furthermore various studies have revealed that herbal medicine (HM) is the most widely used CAM in Africa [7-11]. Sub-Saharan Africa has the highest contribution with 80% of its population relying on HM for therapeutic management of diseases [7].

According to WHO, the term herbal medicine refers to any plant-derived materials or products that contain either raw or processed ingredients from one or more plants with therapeutic or other human benefits [7]. HM is categorized into three types namely: raw plant materials, processed plant materials and medicinal herbal products. Medicinal herbal products comprise of finished, labeled pharmaceutical plant materials [12] and include purified extracts or partially purified active substances that have not been chemically defined. The raw plant materials comprise of crude fresh or dry leaves, flowers, fruits, seeds, roots, barks (stems) of trees, tubers, rhizomes or other plant parts. The processed plant materials are treated according to traditional procedures to improve their safety and efficacy, to facilitate their clinical use, or to make medicinal preparations [12].

In Uganda, use of herbal medicines have been reported as home based remedies for managing various health conditions like malaria [13], eye diseases, diarrhea , fungal infections , common cold, jaundice and skin disorders [14]. Its use has also been reported in the management of: other chronic diseases like HIV/AIDs, liver fibrosis [15], diabetes 2 [16], in pregnancy [17], abortions [18], breast cancer patients [19] and managing sexual impotence [20] amongst others. Although there are anecdotes of its use among SCD patients, there is no published literature on the use of HM in the management of children with SCD in Uganda.

Despite the advancements in the conventional treatment of SCD that has led to introduction of newer drugs like Hyroxyurea, access to such modern treatments remains limited in developing countries like Uganda [10,21]. The high costs associated with conventional medical treatment coupled with high levels of poverty, spiritual and traditional beliefs make HM use a very attractive alternative.

The use of herbal medicine has a great potential to impact on the health care-seeking behavior of patients as patronage of HM may lead to patient´s withdraw from seeking conventional treatment [16]. Although this has not been documented at MSCC, there are anecdotal reports that a number of patients withdraw from attending the routine follow up visits at the Mulago sickle cell clinic. This has to some extent been attributed to home based use of herbal medication which results to patients terminating/interrupting conventional treatment thus leading to high morbidity. In addition, concurrent use of HM and conventional medicine may expose the patients to adverse effects arising from drug interactions [22-24].

We hoped that this study would raise awareness among health care providers at MSCC to the existing use of herbal medicine among their clients. Knowledge of the extent of HM use could also ignite keen interest and therefore studies on the various herbal medicines used by care givers so that their safety and efficacy is well assessed.

## Methods

### Study design

This was a descriptive cross-sectional study that was conducted using a quantitative approach for data collection.

### Study setting

This study was conducted at Mulago Hospital sickle cell clinic (MSCC). MSCC is located at Mulago national referral hospital in Kampala (MNRH). It´s the largest center for specialized SCD diagnosis, research and comprehensive treatment of SCD patients in Uganda. It receives a large number of SCD patients referred from around Uganda and East Africa. This is also the only national specialized referral SCD clinic in Uganda. It handles over 1,000 patients a month with 50 - 70 patients reporting a day. These include new cases and those reporting for routine follow-up and treatment from the clinic. Patients that are unstable by time of closure are referred for admission at the Pediatric emergency ward also called Acute care unit at Mulago National referral hospital -MNRH for admission.

### Study population

The study target population included all caregivers that sought health care services for their children with sickle cell disease from MSCC during the study period. A total of 384 caregivers were recruited for the study using a convenient consecutive sampling technique.

### Inclusion/exclusion criteria

The study recruited care takers of children that were diagnosed of SCD and were seeking care from Mulago Sickle cell clinic. These were both male and female above 18 years of age, staying and caring for a child with SCD child and who willingly consented to participation in the study. Upon identification, potential respondents were approached for consent and requested to complete the study questionnaire which took about 15 minutes of their time to complete. Caretakers of critically ill patients were excluded so as not to delay care. A caretaker would also be excluded in case they couldn´t speak English or Luganda to avoid costs of hiring interpreters.

### Study variables

The dependent variable of the study was the use of HM by care givers of children with SCD. This was determined by asking the caregiver whether he/she has ever used HM in the management of their child with SCD. The answer to this question was coded as Yes=1 and No=0, where use means current use or ever used HM. The independent variables in this study were the various socio-demographic factors, personal based factors, health service related factors and community based factors that influenced the use of HM in the management of children with SCD.

### Data collection tools

Data was collected using a semi-structured researcher administered questionnaire that was developed with input from the studies in the literature review. Key aspects of data included socio-demographics characteristics of the participants, history of past and present use, and factors associated to use of the herbal medicines. The questionnaire was pretested to determine the time required for completion and included both open and close ended questions. Other key aspects considered included ensuring the correct phrasing and sequencing of questions. Two research assistants were recruited and trained on how to administer the questionnaires. The study was explained to caretakers and only those that agreed to participate were recruited. The questionnaires were administered face to face and in the waiting room where the vital signs of the children are measured.

### Data quality control

The questionnaires were pretested on 10 caregivers at another pediatrics ward within the same hospital to assess the flow of the questions. Two research assistants were recruited and trained on how to administer the questionnaires. The questionnaires were administered by face to face interviews to ensure completeness of the questionnaires. Entry of data and analysis was done by the principle investigator to ensure accuracy. The participants were assured of confidentiality with which the data will be handled with and they were encouraged to honestly respond to the questions in the tool.

### Data management and analysis

All the questionnaires were kept safely under key and locked; they were inspected for completeness and consistency. Any questionnaire with less than 90% response was eliminated. Data was coded and entered into SPSS version 23 software. Descriptive analysis was conducted and median and mean computed for continuous variables. Bivalent analysis was done and chi - square tests were conducted to gauge the association between dependent and independent variables. Associations are reported using odds ratios and 95% confidence intervals, level of significance was taken to be a probability value of less than 0.05. Factors found to be significant at bivariate level were entered into a binary logistic regression model for multivariate analysis.

### Ethical consideration

Ethics approval was obtained from the Makerere University School of Health Sciences Research and Ethics Committee and administrative clearance obtained from Mulago Hospital Research and Ethics Committee before study commencement. Participants were adequately informed in a language that they understood about the nature, expectations, potential benefits and risk of the study. Informed consent was sought before participants were enrolled into the study.

## Results

### Socio-demographic characteristics of study participants

A total of 384 caretakers whose children have sickle cell disease and attend follow-up clinics at MSCC were interviewed 295 (76.8%) of them being females. The median age of the participants was 32 years and ranged from 18 - 75years. Two hundred ninety-six (77.1%) respondents were Christians and majority (70.8%) identified themselves as married or cohabiting (Table 1).

**Table 1:** socio-demographic characteristics of the respondents

Characteristic	Frequency (N = 384)	Percentage (%)
**Age (years)**		
18 - 32	194	50.5
33 - 75	190	49.5
**Gender**		
Male	89	23.2
Female	292	76.8
**Marital status**		
Single	63	16.4
Married/cohabiting	272	70.8
Separated	32	8.4
Widowed	17	4.4
**Ethnicity**		
*Baganda	231	60.2
Basoga	33	8.6
Banyankole	21	5.5
Batooro	13	3.4
Iteso	10	2.6
Others	76	19.7
**Religion**		
Christians	296	77.1
Muslims	88	22.9
**Education level**		
Primary and below	110	28.6
Secondary and beyond	274	71.4
**Residence**		
Rural	101	26.3
Urban	283	73.7
**Occupation**		
Housewife	70	18.2
Farmers	34	8.9
Businessman/woman	134	33.3
Professional job	50	13.0
Vocational job	73	19.0
No job	29	7.6
**Earning (Ug. shs)**		
≤ 200,000	206	53.6
>200,000	178	46.4

* Baganda are the commonest ethnic tribe in the central region of Uganda where Mulago Hospital is also located

### Prevalence of herbal medicine use by caregivers of children with sickle cell disease

Of all the participants, only 86 (22.4%) had never used any form of herbal medicine in the management of their children with SCD. Two hundred ninety-eight (77.6%) had ever used some form of herbal medicine for managing a child with SCD. Of these, 235 (61.2%) were currently using herbal medicine. The prevalence reported in this study is the highest being reported from Africa in the recent times. This indicates that there is wide spread use of HM in the management of SCD in Uganda.

### Social-demographic factors associated with use of herbal medicine in children with sickle cell disease

The bivariate analysis of the social-demographic factors associated with the use of herbal medicine by caretakers for their SCD children revealed that none was significantly associated with use of herbal medicines (Table 2). Although, a number of perceptions, community based and health service related factors were found to have associations with the use of HM at bivalent analysis, only those that had a p value < 0.05 were considered significant. This study has revealed that perceptions that herbal medicine cures symptoms faster than Complementary Medicine, HM having fewer side effects than HM and the use of television adverts in marketing herbal medicine have significant impact on the use of HM.

**Table 2:** bivariate analysis of socio-demographic factors associated with herbal medicine use among caretakers of children with sickle cell disease attending Mulago sickle cell clinic

Variable	Ever used HM N (%), (N = 298)	Never used HM, N (%), N = 86	P-value	Cured Odds Ratio (95% CI)
Gender*				
Male	67 (75.3)	22 (24.7)	0.549	0.844 (0.484 – 1.471)
Female	231 (78.3)	64 (21.7)		
**Age (years)**				
18 – 32	150 (77.3)	44 (22.7)	0.892	0.967 (0.599 – 1.563)
33 - 75	148 (77.9)	42 (22.1)		
**Religion**				
Christians	225 (76.0)	71 (24.0)	0.170	0.651 (0.352 – 1.206)
Muslims	73 (83.0)	15 (17.0)		
**Marital status**				
Un married	86 (76.8)	26 (23.2)	0.805	0.936 (0.554 – 1.581)
Married	212 (77.9)	60 (22.1)		
**Level of education**				
Primary and below	85 (77.3)	25 (22.7)	0.921	0.974 (0.574 – 1.653)
Secondary and above	213 (77.7)	61 (22.3)		
**Occupation**				
Employed	75 (75.8)	24 (24.2)	0.609	0.869 (0.507 – 1.489)
unemployed	223 (78.2)	62 (21.8)		
**Level of income**				
≤ 200,000	163 (79.1)	43 (20.9)	0.442	1.207 (0.747 – 1.952)
≥ 200,001	135 (75.8)	43 (24.2)		
**Residence**				
Rural	81 (80.2)	20 (19.8)	0.466	1.232 (0.702 – 2.160)
Urban	217 (76.7)	66 (23.3)		

* Female caretakers/ attendants more commonly bring the sick children to hospitals in our setting.

### Individual, community, and health service related factors associated with herbal medicine use among caretakers of children with sickle cell disease

The bivariate analysis of individual, community and health service related factors (Table 3) revealed that the odds of using HM among caretakers who believed that HM cured symptoms faster than CM were almost 4 times those of caregivers who disagreed with the statement (COR: 3.932, 95% CI: 2.002 - 7.725). The odds of using HM among caretakers who believed that HM had fewer side effects than CM were almost 5 times those of caretakers who disagreed with the statement (COR: 4.931, 95% CI: 2.930 - 8.300). The odds of using HM among care takers who believed that HM improves the lives of the SCD children more than CM were almost 3 times those who didn´t believe the same way (COR: 2.850, 95% CI: 1.558 - 5.216). The odds that a caretaker who agrees that HM manages pain associated with SCD faster than CM using HM were more than 3 times those who disagreed with it (COR: 3.465, 95% CI: 1.529 - 7.851). The odds that a caretaker who agrees that CM works better when used together with HM uses it (HM) for their SCD child were twice those of caretakers that disagreed with the statement (COR: 2.040, 95% CI: 1.251 - 3.328). The odds that a care taker who easily accessed information on HM uses it for their child were almost 2 times those of caretakers who did not access information easily (COR: 1.762, 95% CI: 1.059 - 2.933). The odds that a care taker who agreed that advertising HM on televisions encouraged HM uses it for their child were almost 4 times those their counterparts that disagreed with the statement (COR: 3.586, 95% CI: 2.144 - 5.998). The odds of using HM for a care taker who agreed that HM was available in their communities were 2 times those of caretakers that disagreed with the statement (COR: 2.199, 95% CI: 1.290 - 3.747). The odds that a care taker who agrees that vendors of HM have good communications skills uses HM for their SCD were almost 2 times those heir counterparts that disagreed with the statement (COR: 1.958, 95% CI: 1.134 - 3.382). These findings indicate that there is a close association between the perceptions of caregivers on Herbal Medicine and its use in the management of children with SCD.

**Table 3:** bivariate analysis of individual, community, and health service related factors associated with herbal medicine use among caretakers of children with sickle cell disease

Variable		Ever used HM, N (%) N = 298	Never used HM, N (%) N = 86	P-value	Cured Odds Ratio (95% CI)
HM cures symptoms faster than CM	Agree	109 (90.8)	11 (9.2)	0.000*	3.932 (2.002 – 7.725)
	Disagree	189 (71.6)	75 (28.4)		
HM has fewer side effects	Agree	203 (88.6)	26 (11.4)	0.000*	4.931(2.930-8.300)
	Disagree	95 (61.3)	60 (38.7)		
HM is more effective in treating SCD	Agree	89 (83.2)	18 (16.8)	0.103	1.609 (0.905 – 2.861)
	Disagree	209 (75.5)	68 (24.5)		
HM improves lives of SCD children	Agree	112 (88.2)	15 (11.8)	0.000*	2.850 (1.558 – 5.216)
	Disagree	186 (72.4)	71 (27.6)		
HM is manages pain more effectively	Agree	70 (90.9)	7 (9.1)	0.002*	3.465 (1.529 – 7.851)
	Disagree	228 (74.3)	79 (25.7)		
CM works better when use together with HM	Agree	206 (82.1)	45 (17.9)	0.004*	2.040 (1.251 – 3.328)
	Disagree	92 (69.2)	41 (30.8)		
HM is cheaper	Agree	184 (79.0)	49 (21.0)	0.425	1.219 (0.749 – 1.983)
	Disagree	114 (75.5)	37 (24.5)		
HM is easier to access information on use	Agree	223 (80.5)	54 (19.5)	0.028*	1.762 (1.059 – 2.933)
	Disagree	75 (70.1)	32 (29.9)		
Costs of CM make people to use HM	Agree	220 (79.7)	56 (20.3)	0.114	1.511(0.904 – 2.524)
	Disagree	78 (72.2)	30 (27.8)		
Television adverts encourage HM	Agree	242 (83.7)	47 (16.3)	0.000*	3.586 (2.144 – 5.998)
	Disagree	56 (58.9)	39 (41.1)		
Cultures influence HM	Agree	205 (80.1)	51 (19.9)	0.100	1.513(0.922 – 2.482)
	Disagree	93 (72.7)	35 (27.3)		
HM is readily available	Agree	242 (80.9)	57 (19.1)	0.003*	2.199 (1.290 – 3.747)
	Disagree	56 (65.9)	29 (34.1)		
Radio adverts encourage HM use	Agree	225 (79.2)	59 (20.8)	0.199	1.410 (0.833 – 2.388)
	Disagree	73 (73.0)	27 (27.0)		
Drug shortages at the clinic encourage HM use	Agree	210 (78.7)	57 (21.3)	0.457	1.214 (0.728 – 2.025)
	Disagree	88 (75.2)	29 (24.8)		
Health workers’ poor communication encourages HM use	Agree	104 (80.0)	26 (20.0)	0.420	1.237 (0.737 – 2.077)
	Disagree	194 (76.4)	60 (23.6)		
Long waiting times at clinic encourages HM use	Agree	147 (81.2)	34 (18.8)	0.109	1.489 (0.914 – 2.426)
	Disagree	151 (74.4)	52 (25.6)		
Vendors’ good communication encourages HM use	Agree	244 (80.3)	60 (19.7)	0.015*	1.958 (1.134 – 3.382)
	Disagree	54 (67.5)	26 (32.5)		

HM = Herbal Medicine, CM = Conventional medicine, * results are statistically significant at p-value of < 0.05

### Multivariate analysis of factors associated with HM use

At multivariate analysis, the variables were entered into a logistic regression model. The factors that remained significant were (Table 4): perception that HM cures symptoms faster than conventional medicine (AOR =3.439, 95% CI: 1.447 - 8.176), HM has fewer side effects than CM (AOR =3.528, 95% CI: 1.917 -6.494) and the use of televisions to advertise herbal medicine (AOR =4.185, 95% CI: 2.036 -8.603).

**Table 4:** multivariate analysis of factors associated with herbal medicine use among caretakers of children with sickle cell disease attending Mulago sickle cell clinic

Variable	Adjusted Odds Ratio	95% C.I.	P value
Age	0.906	(0.494 - 1.662)	0.750
Gender	0.753	(0.366 -1.549)	0.440
Religion	0.904	(0.442 -1.849)	0.782
Marital	1.176	(0.608 - 2.277)	0.630
Highest Education level	0.681	(0.341 -1.358)	0.275
Job	0.839	(0.368 -1.914)	0.677
Earning	1.277	(0.596 -2.737)	0.530
Residence	1.109	(0.555 -2.217)	0.769
HM cures symptoms faster than CM	3.439	(1.447 - 8.176)	0.005*
HM has fewer side effects	3.528	(1.917 -6.494)	0.000*
HM is more effective in treating SCD	0.628	(0.268 -1.470)	0.283
HM makes life of children better	1.824	(0.816 -4.077)	0.143
HM is more effective in managing pain	1.984	(0.757 - 5.202)	0.163
CM works better when used with HM	1.668	(0.928 - 2.998)	0.087
HM is cheaper than CM	0.810	(0.420 -1.561)	0.529
It is easy to access information on HM use	0.999	(0.494 -2.019)	0.998
CM is costly so people resort to HM	1.235	(0.651 - 2.343)	0.519
TV adverts on HM use encourage HN use	4.185	(2.036 -8.603)	0.000*
Cultures influence HM use	1.491	(0.806 -2.758)	0.203
HM is readily available in communities	2.037	(1.016 -4.084)	0.045
Radio adverts encourage in HM use	0.613	(0.293 -1.283)	0.194
Lack of medicines at the clinic encourages HM use	0.719	(0.369 -1.402)	0.333
Poor communication by H/W encourage HM	0.804	(0.415 - 1.555)	0.516
Long waiting time at the clinic encourages HM use	1.443	(0.775 -2.687)	0.248
Good communication of HM vendors encourages use	1.270	(0.644 -2.508)	0.490

* Results are statistically significant at p-value of < 0.05

## Discussion

Although various studies have reported global increase in Herbal medicine (HM) utilization especially in chronic diseases like SCD, the prevalence in this study is exceptionally high. It is the highest ever reported prevalence of HM use among children with SCD. Previous African studies have reported prevalence ranging from 15% [6] to 62.9% [25]. Much lower rates were reported in the developed world; 9.1% [26], 3% [27] and 2.5% [28].

This implies that HM is much used as a complementary remedy for children with SCD by majority of the care givers in our setting. Unlike in the developed countries, HM is an integral part of the African healthcare system since time immemorial and this explains the striking difference between the prevalence of HM use in studies conducted in Africa and those from developed countries like USA. The way the outcome variable was measured in this study is the most likely reason for the disparity in prevalence reported in this study and what has been found in previous African studies. In this study, each caretaker was asked if they had ever used any form of HM for managing their child. The prevalence in this study therefore combines those who had ever used HM but stopped and those that were currently using HM for their SCD child unlike the previous studies that focused on current use of HM only. Given that SCD is a chronic condition, associated with various devastating symptoms, the probability that a caretaker had used HM for their SCD child is expected to be high. Indeed several studies have documented that increased use of HM in chronic conditions [6, 28, 29].

Factors associated with herbal medicine use

This study revealed that: perceptions of herbal medicine curing symptoms faster than CM, HM having fewer side effects than CM and the use of television adverts in marketing herbal medicine have significant impact on the use of HM. Although multiple studies have reported HM use basing on its perceived ability to cure symptoms [25,30,31], the perception that it cures symptoms faster than CM has not been previously reported. In support for the above mentioned symptoms cure, previous case control studies have revealed the role of herbal medicine in significantly reducing the levels of sickle blood cells, increasing the concentration of hemoglobin and potent anti-thrombin activity in preventing hypercoagulability state, in SCD [31-34]. The perceptions of faster cure of symptoms with HM would further be strengthened by caretaker dissatisfaction related to access and effects of CM symptoms relapse [30]. However, there is need to probe into its ability to cure these symptoms faster than conventional medicine in our setting.

The findings of beliefs for fewer side effects with HM in this study is similar to what has been reported in other Ugandan and African studies, [35,36]. Whereas many have perceived HM as being safe and devoid of adverse side effects, this may not be totally true as few studies have been conducted to objectively verify it. Ekor (2014) suggests that these perceptions are not only false, but also may be misleading to many of the users. This is possibly due to the fact that many people globally underestimate the toxicity of herbals and never realize that these agents could be as toxic as (or even more than) synthetic drugs [37].This may also be based on misinformation provided by herbalists, relatives and friends that HM are natural foods without any toxic element [35]. Although some studies have backed up the safety of some herbal remedies used in SCD [31,38], there is increasing proof of the interactions of herbal remedies and conventional medicines with potential for serious adverse effects [33,34,37].

Some of the toxicities reported in a systemic review on some HM used for SCD in western Africa included; increased risk for macular rashes, headaches and altered liver function amongst others [31]. Needless to say, none of these were conducted in Uganda. This puts the children using HM at potential risk of acquiring such adverse effects since most of these herbal medicines are often introduced into the market without any safety or toxicological evaluation.

Our results are in agreement with a number of other studies that report the media as the main drivers of the support for HM use [25,36]. In this study, use of television adverts to market HM was observed to significantly influence HM use among children with SCD. Similar findings were observed in a recent qualitative study conducted in Ghana were participants noted that television adverts air attractive videos on HM that influenced its uptake and use [36].

This is a fairly new observation as the impact of TV adverts had not been widely evaluated or reported. Previous media reviews have focused largely on the radio adverts [39]. The television adverts could have increased the impact and as such may have become very significant in our study because most of the participants were urban dwellers that basically rely on televisions as source of information. In addition, televisions air video adverts that are very attractive and designed to be persuasive thus excite and lead many caretakers to seek HM for their children with SCD. The common concerns about media reporting include: unnecessary sensationalism, inadequate follow-through, failure to consider the quality of evidence, inaccurate portrayal of benefits, lack of consideration of adverse effects and costs associated with HM use [40].

### Limitations of the study

We report these as potential limitations: 1) First, we used a 5 level Likert scale to collect data on the factors that influence HM use where respondents had to “strongly agree”, “agree”, “neither agree or disagree”, “disagree”, “strongly disagree”. During categorization of data, participants that were neutral were considered to disagree with the particular question. This may have affected the results because their opinion may not have been correctly represented; nonetheless we only had a few cases of nonresponsive; 2) This was a quantitative study that hence we could not exhaustively explore the various perceptions caretakers reported about HM use. There is need for a qualitative study to explore the various perceptions caretakers have about HM; 3) The consecutive (non-probability) sampling technique employed could limit generalizability, nonetheless it was optimal for this study.

## Conclusion

There is high rate of HM use among our children with SCD and supported by perceptions that HM treats symptoms faster than CM, and is safe. Media and specifically the attractive television adverts have increased its uptake. These need to be checked for authenticity and correctness.

### What is known about this topic

- The use of herbal medicine in the management of sickle cell disease has widely been reported in some of the African countries like Nigeria and Democratic Republic of Congo especially in communities where conventional medication is often not available or too expensive for the locals;
- Some herbal products like Niprisan® and Ciklavit® have been studied and appeared to be safe and effective in reducing severe painful crises.

### What this study adds

- To the best of our knowledge, there is no published data on the prevalence and factors associated with herbal medicine in the management of sickle cell disease in Uganda. This study provides evidence and prevalence of herbal medicine use by care givers of children with sickle cell disease in Uganda. With this evidence, further studies should be conducted to identify the specific herbals being used and assess their safety and effectives in the management of the disease;
- The study has also documented the factors influencing use of herbal medicine in the management of children with SCD in Uganda.
